# *SUMF1* Common Variant rs793391 Is Associated with Response to Inhaled Corticosteroids in Patients with COPD

**DOI:** 10.3390/ijms262010225

**Published:** 2025-10-21

**Authors:** Charikleia Ntenti, Eleni Papakonstantinou, Leticia Grize, Maria Pascarella, Björn C. Frye, Sebastian Fähndrich, Despoina Ioannidou, Spasenija Savic Prince, Antonis Goulas, Daiana Stolz

**Affiliations:** 1 Clinic of Respiratory Medicine, University of Freiburg, Faculty of Medicine, University of Freiburg, 79098 Freiburg im Breisgau, Germany; charnten@auth.gr (C.N.); bjoern.christian.frye@uniklinik-freiburg.de (B.C.F.); sebastian.faehndrich@uniklinik-freiburg.de (S.F.); 2 First Laboratory of Pharmacology, School of Medicine, Aristotle University of Thessaloniki, 54124 Thessaloniki, Greece; 3 Emergency Department, University Hospital Basel, 4031 Basel, Switzerland; 4 Pulmonary Department, Hippokration General Hospital of Thessaloniki, 54642 Thessaloniki, Greece; 5 Pathology, Institute of Medical Genetics and Pathology, University Hospital Basel, University of Basel, 4031 Basel, Switzerland; 6 Clinical Research Unit, Special Unit for Biomedical Research and Education, School of Medicine, Aristotle University of Thessaloniki, 54124 Thessaloniki, Greece

**Keywords:** COPD pharmacogenetics, single nucleotide polymorphism, sulfatase modifying factor 1, inhaled corticosteroids, sulfatases, precision medicine, personalized therapy

## Abstract

This study investigated whether specific *sulfatase modifying factor-1* (*SUMF1*) SNPs—previously linked to lung function—are associated with COPD progression and response to inhaled corticosteroid (ICS) treatment, specifically budesonide, given that *SUMF1* expression is altered in COPD and its variants linked to increased disease risk. A subgroup of 165 COPD patients from the HISTORIC study were genotyped for two common *SUMF1* SNPs, rs11915920 and rs793391. Patients first underwent a six-week run-in phase with open-label triple inhaled therapy (LAMA/LABA/ICS), then were randomized to receive either LAMA/LABA/placebo or LAMA/LABA/ICS for 12 months. Associations between SNPs, baseline characteristics, and response to ICS—based on FEV_1_ change over 12 months—were evaluated. Heterozygotes (TG) for the rs793391 polymorphism treated with LAMA/LABA/ICS showed a significant and clinically meaningful FEV_1_ improvement compared to the placebo group. This was supported by improved patient-reported outcomes, with lower SGRQ and CAT scores and a clinically relevant increase in General Health Questionnaire scores. These findings suggest that rs793391 may be linked to both COPD progression and ICS response and could contribute to more personalized treatment strategies in COPD.

## 1. Introduction

Recent investigations have focused on identifying the genetic underpinnings of Chronic Obstructive Pulmonary Disease (COPD) to improve our understanding of the disease and its progression. Using genome-wide association studies (GWAS) and whole-genome sequencing, researchers have identified various genes and single nucleotide polymorphisms (SNPs) associated with COPD [1,2,3]. Inhaled corticosteroids (ICS) are the most used and effective treatment for asthma [4]. However, COPD patients often do not respond as well to ICS, likely due to genetic factors. Furthermore, glucocorticoid unresponsiveness in COPD hampers effective treatment [5]. ICS in COPD patients unresponsive to treatment may lead to increased risks of pneumonia, oral thrush, increased exacerbations, and systemic side effects without improving outcomes [6,7,8,9]. Long-term ICS use also increases the risk of pneumonia, oral candidiasis, and dysphonia [10]. Therefore, understanding the genetic factors influencing the ICS response in COPD patients could enable physicians to personalize treatment decisions and action plans for individual patients.

Sulfatases are enzymes that modify proteoglycan chains through the removal of sulfate groups and are, thus, critical for extracellular matrix (ECM) remodeling. All sulfatases are regulated by the sulfatase modifying factor 1 gene (*SUMF1*), several polymorphisms of which have been associated in the past with predisposition to COPD [11]. *SUMF1*, the protein product of *SUMF1*, displays a formylglycine-generating enzymatic activity catalyzing a post-translational modification of sulfatases required for full activity (11). Mutations in *SUMF1* are a leading cause of a variety of human diseases, many of them causing detrimental effects in the lungs [12]. It is well-established that the impaired expression of *SUMF1* can cause lung function decline by affecting alveoli, related to an overabundance of sulfated glycosaminoglycans (GAGs) deposited in the ECM [13]. Thus, *SUMF1* polymorphisms could conceivably be associated with lung function and clinical characteristics of COPD patients [14]. In the present study, we explore the association of two *SUMF1* common single nucleotide polymorphisms (SNPs) with lung function characteristics, histological and biochemical parameters, and response to therapy based on annual FEV1 change and health-related quality of life (QoL) data in a group of patients with COPD.

## 2. Results

### 2.1. Patients’ Characteristics, Genotype and Allele Frequencies

The study involved 165 Caucasian patients, 31.1% of whom were females, with a median age of 67 years (range 59–72 years). These patients had a smoking history of an average of 50 pack-years (range 37–70 pack-years) and had been living with the disease for an average of 60 months (range 23–120 months). Their post-bronchodilator FEV1% predicted value was 59% (range 49–67%), and their post-bronchodilator diffusing capacity for carbon monoxide (DLCO SB)% predicted value was 61.5% (range 48–78%) (Table 1).

Table 2 displays the genotype and allele frequencies of the rs11915920 and rs793391 polymorphisms in the *SUMF1* gene. The genotype distributions were consistent with the Hardy–Weinberg equilibrium, as indicated by both *p* values being greater than 0.88 (Table 2). LD analysis showed that rs11915920 and rs793391 are not in strong linkage disequilibrium (r^2^ = 0.145, D = –0.39), consistent with their independent distribution in our cohort.

### 2.2. Association of SUMF1 Polymorphisms with Lung Function, Histological and Blood-Sampling Parameters at Baseline

There were no significant associations of the rs11915920 and rs793391 polymorphisms with any of the lung function parameters at baseline (Supplementary Table S2).

Patients homozygous for the *SUMF1* rs11915920 (TT) exhibited a higher eosinophil tissue infiltration (*p* = 0.006) (Supplementary Table S3). Rs11915920 heterozygotes (CT) had significantly lower values of ASMC area, assessed in endobronchial biopsies (*p* = 0.015) (Supplementary Figure S1 and Table S3).

Patients heterozygous for the *SUMF1* rs11915920 (CT) presented with higher blood neutrophils (*p* = 0.013) (Supplementary Table S4). Blood eosinophils were marginally higher in patients homozygous for the reference allele (TT) of *SUMF1* rs793391 (*p* = 0.050) (Supplementary Table S4).

### 2.3. Association of SUMF1 Polymorphisms with Response to Treatment with ICS

**Figure 1:**
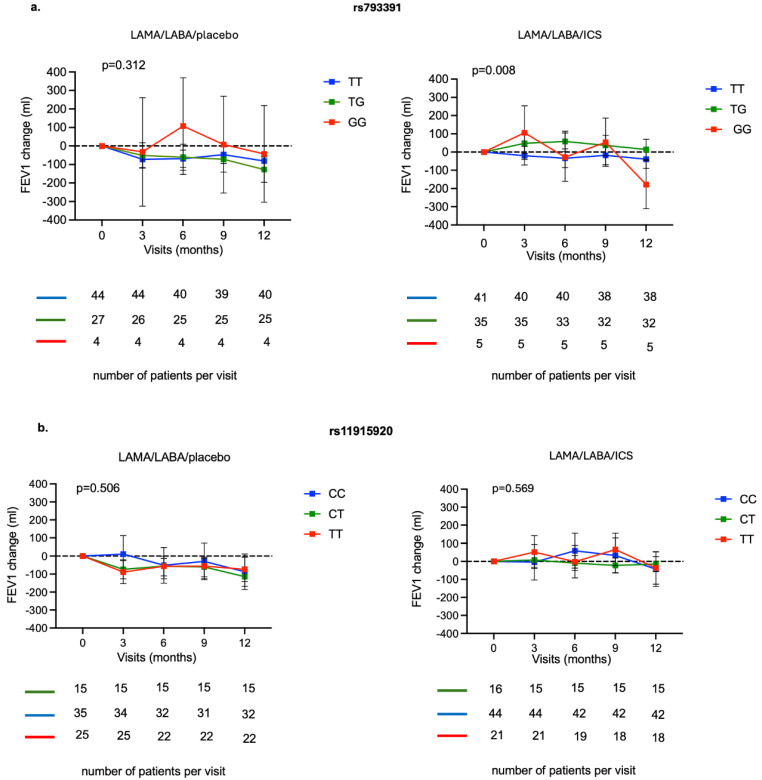
FEV1 change (mL) over the study visits during 12 months for patients randomized to LAMA/LABA/placebo and LAMA/LABA/ICS for rs793391 (**a**) and rs11915920 (**b**) genotypes. The association between FEV1 change from visit 1 in the treatment groups and different visits was evaluated using a mixed-effects model. The treatment group and visit number (categorical factors) were included as fixed effects and the subject as a random effect. The Dunnett–Hsu method was used to correct *p*-value for multiple comparisons. To calculate the change from visit 1 to visit 5 for treatment group, an analysis of covariance was used.

FEV1 change over 12 months was similar in all genotypes of the rs793391 and rs11915920 polymorphisms when patients were receiving LAMA/LABA/placebo (Figure 1a,b). However, for COPD patients receiving LAMA/LABA/ICS there was a significant impact of the rs793391 polymorphism on FEV1 change over 12 months (*p* = 0.008) (Figure 1a).

**Figure 2:**
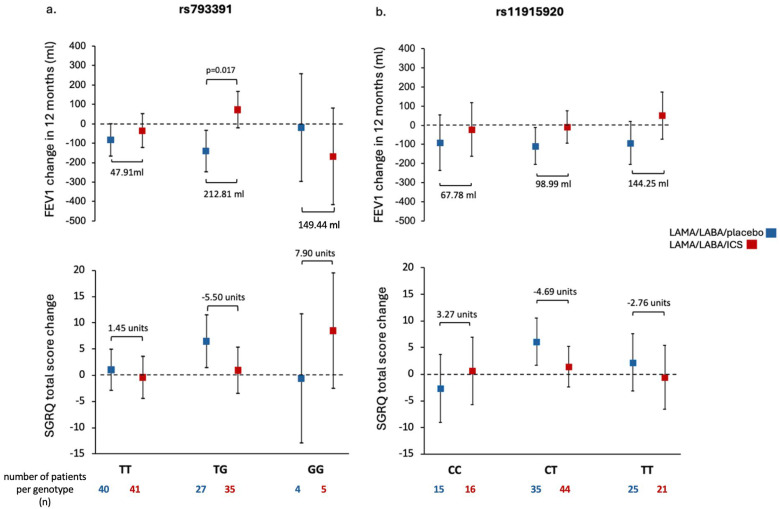
Change in FEV1 (mL) and change in SGRQ over 12 months in COPD patients stratified according to rs793391 (**a**) and rs11915920 (**b**) genotypes. The squares represent the mean and the whiskers 95% CI. Two-step regression models were used to evaluate the change in FEV1 from the first visit (after 6 weeks run-in period with triple therapy) to the last visit, 12 months after randomization, in the treatment groups. In the first step, the FEV1 change in each patient was calculated as the slope of a linear regression of the five FEV1 measurements over time. In the second step, the slopes representing the change in FEV1 were regressed in function of the treatment groups. A generalized linear regression of the change in SGRQ total score from the first visit (after 6 weeks run-in period with triple therapy) to the last visit, 12 months after randomization, in the treatment groups is presented. Total scores on the SGRQ range from 0 to 100, with lower scores indicating better health-related quality of life. A decrease in the SGRQ total score of ≥4 units indicate a clinically relevant response. The Dunnett–Hsu method was used to correct *p*-values for multiple comparisons. Only statistically significant *p*-values are shown.

Further analysis between the treatment groups revealed significant differences in FEV1 change over 12 months between patients treated with LAMA/LABA/placebo and those on LAMA/LABA/ICS across the various genotypes of rs793391. Heterozygous individuals (TG) receiving LAMA/LABA/ICS demonstrated a significant, mean increase in FEV1 of 71.8 mL (95% CI: −22.1–165.6 mL) after 12 months of treatment, that is, 212.8 mL more compared to patients who were given LAMA/LABA/placebo (95% CI: −247.9–34.2 mL) (*p* = 0.017). Patients homozygous for the alternative allele (GG) who underwent triple therapy (LAMA/LABA/ICS) exhibited a decrease in FEV1, with an adjusted mean loss of 149.44 mL compared to patients treated with LAMA/LABA/placebo (Figure 2a).

Consistent with the previously presented FEV1 changes, TG carriers of the rs793391 genotype who received LAMA/LABA/ICS treatment showed a mean, clinically meaningful reduction of 5.5 units in the SGRQ total score compared to those with the same genotypes receiving LAMA/LABA/placebo over 12 months (Figure 2a). Although statistical significance was not reached, similar trends were observed in the CAT and SF-36 General Health scores: TG carriers treated with LAMA/LABA/ICS tended to have lower, mean CAT scores (−2.68 units) and higher, mean SF-36 scores compared with those on LAMA/LABA/placebo, suggesting a potential benefit of triple therapy in this genotype subgroup (Table 3 and Table 4).

To explore the genetic architecture of rs793391 and ICS responsiveness, both dominant and over-dominant inheritance models were assessed. In the dominant model (TT vs. TG + GG), ICS treatment was associated with a statistically significant improvement in FEV_1_ slope (*p* = 0.004) (Supplementary Figure S2a) and absolute change (mL) in carriers of the G allele (*p* = 0.038) (Supplementary Figure S3a). However, this effect appeared to be driven primarily by heterozygous TG individuals, as the number of GG homozygotes was very small (*n* = 9) and insufficient to influence the model independently.

**Figure 3:**
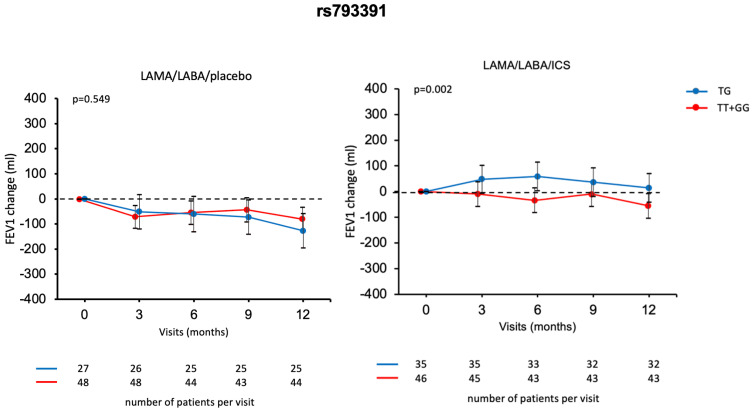
FEV_1_ change (mL) over the study visits during 12 months for patients randomized to LAMA/LABA/placebo and LAMA/LABA/ICS by rs793391 over-dominant (TG vs. TT + GG) genotype model. The association between FEV_1_ change from visit 1 in the treatment groups and different visits was evaluated using a mixed-effects model. The treatment group and visit number (categorical factors) were included as fixed effects and the subject as a random effect. The Dunnett–Hsu method was used to correct *p*-value for multiple comparisons. To calculate the change from visit 1 to visit 5 for treatment group, an analysis of covariance was used.

To further explore the association between rs793391 and ICS response, we applied an over-dominant model (TG vs. TT + GG). As shown in Figure 3, this model confirmed the findings from the main analysis and revealed a significant treatment effect for the ICS group. Specifically, TG carriers exhibited a significantly greater improvement in FEV_1_ over the 12-month period when treated with LAMA/LABA/ICS compared to LAMA/LABA/placebo (*p* = 0.002). No significant differences were observed between the genotype groups in the placebo arm (*p* = 0.549), suggesting that the beneficial effect of ICS was specific to the TG genotype.

**Figure 4:**
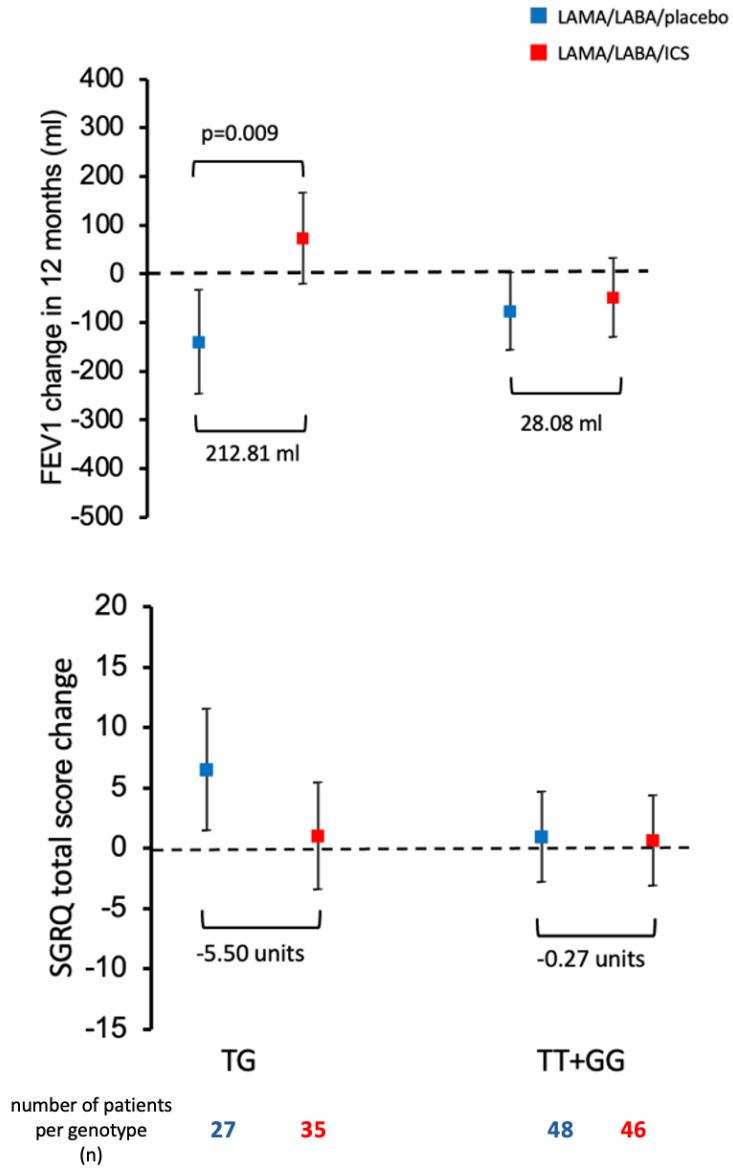
Change of FEV_1_ (mL) and change in SGRQ over 12 months in COPD patients stratified according to rs793391 (TG vs. TT + GG) over-dominant genotype model. The squares represent the mean and the whiskers 95% CI. Two-step regression models were used to evaluate the change of FEV_1_ from the first visit (after 6 weeks run-in period with triple therapy) to the last visit, 12 months after randomization, in the treatment groups. In the first step, the FEV_1_ change in each patient was calculated as the slope of a linear regression of the five FEV_1_ measurements over time. In the second step, the slopes representing the change in FEV_1_ were regressed as a function of the treatment groups within the over-dominant genotype categories (TG vs. TT + GG). A generalized linear regression of the change in SGRQ total score from the first visit (after 6 weeks run-in period with triple therapy) to the last visit, 12 months after randomization, in the treatment groups was also performed. Total scores on the SGRQ range from 0 to 100, with lower scores indicating better health-related quality of life. A decrease in the SGRQ total score of ≥4 units indicates a clinically relevant response. The Dunnett–Hsu method was used to correct *p*-values for multiple comparisons. Only statistically significant *p*-values are shown.

Consistent with the trajectory plots, slope-based regression analysis (Figure 4) further demonstrated that TG carriers in the ICS group had a statistically significant increase in FEV_1_ over time compared to TT + GG carriers. Additionally, a meaningful reduction in SGRQ total score was observed for TG carriers receiving ICS, exceeding the threshold for clinically relevant improvement (≥4 units). These findings support a genotype-specific response to ICS treatment, both in terms of lung function and health-related quality of life.

The recessive model (GG vs. TT + TG) did not yield statistically significant associations for FEV_1_ or SGRQ outcomes, which is not surprising given the very small number of GG homozygotes in our cohort (*n* = 9, 4 and 5 per treatment group), precluding meaningful analysis (Supplementary Figures S2b and S3b).

The response to treatment in patients with various genotype combinations was further explored. Combinations of SNPs were considered only if each combination had five or more patients for each treatment group. Heterozygotes for both SNPs (CT/TG) exhibited a significantly better response (*p* = 0.039) to treatment with LAMA/LABA/ICS (median improvement 17.1 mL, IQR: −96.4–128.3 mL) compared with patients with the same genotypes who received LAMA/LABA/placebo (median loss of 59.4 mL, IQR: −172.9–−35.1 mL) (Supplementary Figure S2).

To explore potential genotype-specific effects on exacerbation risk, adjusted annual AECOPD exacerbation rates were analyzed in relation to rs793391 genotype and treatment allocation. Overall, exacerbation rates were relatively low in both treatment arms, which may limit statistical power and the reliability of comparisons. Among patients in the ICS group, TG carriers exhibited the lowest exacerbation rate (0.61 [95% CI: 0.30–0.92]), with a statistically significant difference compared to GG carriers (1.67 [95% CI: 0.85–2.49], *p* = 0.036), while the difference compared to TT carriers (0.76 [95% CI: 0.48–1.05]) was not significant (*p* = 0.720). In the placebo group, no significant differences in exacerbation rates were observed across genotypes: TG carriers had a rate of 1.05 (95% CI: 0.65–1.45), compared to 0.87 (95% CI: 0.55–1.18) in TT carriers (*p* = 0.711) and 0.46 (95% CI: –0.58–1.49) in GG carriers (*p* = 0.483) (Supplementary Table S5).

COPD patients carrying the rs11915920 variant and receiving LAMA/LABA/ICS showed a mean improvement in FEV_1_ over 12 months of 68 mL, 99 mL, and 144 mL for CC, CT, and TT genotypes, respectively, compared with patients receiving LAMA/LABA/placebo. Among these, only TT carriers treated with ICS demonstrated a net gain in FEV_1_; however, this improvement did not reach statistical significance (Figure 2b). In terms of patient-reported outcomes, only heterozygous CT carriers receiving LAMA/LABA/ICS showed a marginally clinically relevant improvement, with a mean reduction of 4.7 units in SGRQ total score compared to placebo (Figure 2b). Additionally, TT carriers showed a trend toward improved general health, with a mean increase of 5.97 units in the SF-36 General Health score. However, no clinically meaningful differences were observed in SGRQ or CAT scores within this subgroup (Table 3 and Table 4).

## 3. Discussion

The present study examined two SNPs, rs11915920 and rs793391, within the *SUMF1* gene that were previously associated with COPD prevalence and altered lung function. Our findings suggest that the rs793391 *SUMF1* polymorphism may influence the response to treatment with ICS, particularly budesonide, combined with LAMA/LABA, in a well-characterized cohort from the HISTORIC study. Additional analyses using different inheritance models, particularly the dominant and over-dominant models, reinforced this observed pharmacogenetic association.

At baseline, there were no significant differences in lung function characteristics among different genotypes of the rs11915920 and the rs793391 polymorphisms. However, a statistically significant and clinically meaningful association emerged between rs793391 and ICS treatment at the end of the 12-month study. TG carriers receiving triple therapy showed a mean FEV1 improvement of 212.8 mL compared to those in the placebo group. To better understand this association, we further explored alternative genetic models. The dominant model (TG + GG vs. TT) revealed statistically significant treatment–genotype interactions, primarily driven by the TG subgroup, as GG carriers were rare (*n* = 9). The over-dominant model confirmed this trend, while the recessive model (GG vs. TT + TG) lacked statistical power due to low sample size and is of limited interpretive value.

In our study, TG carriers of rs793391 (*n* = 35) responded significantly better to LAMA/LABA/ICS treatment compared to the other two genotypes (*n* = 46) who received either triple or dual therapy. This pattern is consistent with a potential heterozygote advantage, where individuals with the TG genotype derive greater therapeutic benefit than either homozygous group. Conversely, GG carriers demonstrated less favorable outcomes when treated with ICS, including a decline in FEV1 and deterioration in health-related quality of life over 12 months, raising the hypothesis that ICS may be detrimental in this genotype. Although limited by the small sample size of this group, these observations warrant further investigation.

In a previous population-based cohort study of both COPD patients and controls, Jarenbäck et al. showed that there is a link of multiple SNPs in *SUMF1* with COPD prevalence, identifying rs793391 as a particularly significant variant [15]. In their study, a group of genetic variations in the *SUMF1* gene were associated with COPD prevalence and differences in lung function parameters such as FEV1 and FEV1/FVC. Specifically, it was found that TG carriers had significantly higher FEV1% predicted and FEV1/FVC ratios compared to homozygous individuals. However, such associations were not replicated in our cohort, likely due to differences in study populations: our study enrolled only moderate-to-severe COPD patients (grades B–D), while theirs included healthy individuals and patients with varying disease severity [15].

We also examined whether rs793391 genotypes influenced exacerbation risk. Overall, the exacerbation rate was low across both treatment arms, likely reflecting the clinical trial setting and stable patient population. Although no statistically significant differences were observed, TG carriers treated with ICS had numerically fewer exacerbations (0.61 vs. 0.76 events/year) than TT carriers, with a *p*-value of 0.06, suggesting a trend that may merit further investigation. Due to the low number of exacerbations and absence of emergency visit data, these findings should be interpreted with caution.

While there is an established association between rs793391 and *SUMF1* expression [16], the specific molecular mechanisms connecting it to COPD are still undefined. Considering its intronic location, it is conceivable that this SNP could serve as a precursor for small RNA molecules, like microRNA, suggesting an avenue for further investigation into its functional role.

Our study has several limitations. First, the absence of a second, independent validation cohort and the relatively small sample size (*n* = 165), which may reduce statistical power and contribute to false negatives. Post hoc power analysis indicated ~80% power to detect a 150 mL difference in FEV1 change, consistent with the minimal clinically important difference (MCID) in COPD [17], but smaller genetic effects may have gone undetected. Second, all patients received budesonide as the ICS component, and our findings may not generalize to other ICS compounds with different pharmacokinetic or pharmacodynamic profiles. Third, the low exacerbation rate and limited number of GG carriers reduce the reliability of some subgroup analyses.

Despite these limitations, the study has important strengths. It is based on a well-characterized randomized cohort, and combines robust clinical outcomes (FEV1, QoL) with genetic data. The results highlight a potential role for pharmacogenetic testing in identifying COPD patients most likely to benefit from ICS therapy.

While the prospect of *SUMF1* genotyping is attractive, practical considerations such as cost, turnaround time, and clinical feasibility must be addressed. Furthermore, validation in larger, ethnically diverse populations is necessary to confirm the utility of these findings. The impact of *SUMF1* on lung physiology is well-established [15,16]. Additionally, the effect of rs793391 on various lung function characteristics is evident, as demonstrated by consistent studies showing its association with lung function and COPD across diverse groups, including healthy active smokers, former smokers, and even never-smokers. Our findings further extend its impact to treatment response with LAMA/LABA/ICS. If independently confirmed, this outcome (i) suggests the clinical usefulness of genotyping the rs793391 polymorphism to predict response to ICS and (ii) underscores the genetic influence on the expression and sulfation pattern of GAGs, highlighting their role in COPD and airway remodeling diseases in general.

Our findings contribute to the expanding field of COPD pharmacogenetics by highlighting a novel association between the *SUMF1* rs793391 polymorphism and clinical response to ICS treatment. While prior studies have largely focused on genes involved in glucocorticoid signaling—such as *GLCCI1* and *FKBP5* [18,19]—with inconsistent replication, limited attention has been given to genes related to lung structure and tissue remodeling. In our recent review [20], we underscored the lack of validated pharmacogenetic markers in COPD and the challenges in translating such findings into clinical practice. By investigating *SUMF1*, a gene previously linked to COPD risk and altered lung expression, this study addresses a key gap.

In summary, our results point to a potential pharmacogenetic marker for ICS responsiveness in COPD, although the findings remain exploratory and hypothesis-generating. The observed genotype–treatment interactions on lung function trajectories and FEV_1_ changes are promising but should be interpreted with caution. Further validation in larger, independent cohorts with longer follow-up is needed to confirm the reproducibility, clinical relevance, and feasibility of incorporating *SUMF1* genotyping into routine COPD management.

## 4. Materials and Methods

### 4.1. Study Population

**Figure 5:**
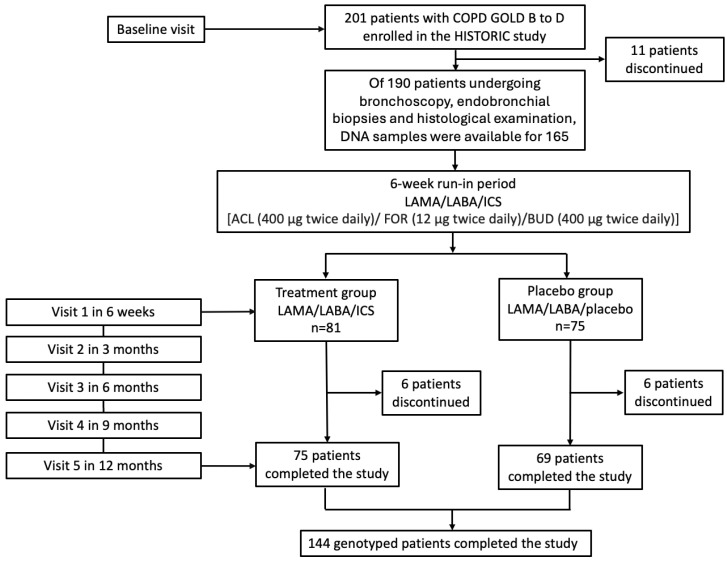
Pharmacogenetic study flowchart: Of the 201 patients enrolled in the HISTORIC study, 11 patients discontinued before bronchoscopy. Among the 190 participants who underwent bronchoscopy, endobronchial biopsies, and histological examination, DNA samples were available for 165 individuals, enabling pharmacogenetic analysis. Of these patients, 156 participated in the initial visit, and ultimately, 144 completed the study. GOLD: Global Initiative for Chronic Obstructive Lung Disease.

The study included 165 patients diagnosed with COPD grades B-D as per the Global Initiative for Chronic Obstructive Lung Disease (GOLD) guidelines, who were participants in the HISTORIC study, an investigator-initiated, double-blind, randomized, placebo-controlled trial conducted at the University Hospital of Basel, Switzerland [21]. Among the 190 participants who underwent bronchoscopy, endobronchial biopsies, and histological examination, DNA samples were available for 165 individuals, enabling pharmacogenetic analysis. Of these patints, 156 participated in the initial visit, and ultimately, 144 completed the study. Importantly, there was no evidence of selection bias in the availability of DNA samples, as the subset of 165 participants was representative of the overall study population in terms of key demographic and clinical characteristics (Figure 5).

Patients followed a 6-week run-in period on an open-label triple inhaled therapy consisting of aclidinium/formoterol/budesonide (ACL/FOR/BUD: 400/12/400 mcg/bid), after which they were randomized to receive either ACL/FOR/BUD or ACL/FOR/Placebo for 12 months. Ethical approval was granted by the institutional review board (EKNZ 2016-6-01880), and the trial was registered on 15/11/2016 with the International Standard Randomised Controlled Trial Number (ISRCTN11017699) [22], adhering to the Declaration of Helsinki and good clinical practice guidelines. Written informed consent for the analysis was obtained from all participants.

### 4.2. Sample Size and Power Justification

No formal a priori sample size calculation was performed for this pharmacogenetic sub-analysis, as it was conducted on the subset of 165 patients from the HISTORIC trial with available DNA samples. A post hoc power assessment indicated that, with approximately 82 patients in the LAMA/LABA/ICS group and 83 in the LAMA/LABA/placebo group, the study had ~80% power to detect a between-group difference in FEV_1_ change of 150 mL, assuming a standard deviation of 200 mL and a two-sided α = 0.05. This detectable difference aligns with the MCID commonly reported in COPD clinical trials [17].

### 4.3. DNA Extraction and Genotyping

Genomic DNA was extracted from the peripheral blood of participants using the QIAsymphony DSP DNA Kit (QIAGEN, Hilden, Germany), following the manufacturer’s protocol, and stored at −80 °C until genotyping. Genotyping of all SNPs was performed using real-time PCR and TaqMan chemistry, with probes designed via Primer Express software and produced by Applied Biosystems (ABI, Warrington, UK). Data analysis was conducted using SDS 2.0 software (ABI, Foster City, CA, USA).

### 4.4. Linkage Disequilibrium Analysis

To evaluate whether the two SNPs represent independent genetic signals, rs11915920 and rs793391, a linkage disequilibrium (LD) analysis was performed using binary presence of minor alleles (T for rs11915920 and G for rs793391) across the 165 genotyped individuals. LD estimates, including D and r^2^, were calculated from a 2 × 2 allele presence matrix using statsmodels (version 0.14.0) in Python.

### 4.5. Statistical Analysis

The study data were summarized using counts and percentages or medians with interquartile ranges. Categorical data were compared using Fisher’s exact test, and continuous data were analyzed using the Kruskal–Wallis test. The association between FEV1 change for each genotype from visit 1, treatment group, and visit number was assessed using a mixed-effects model. The treatment group and visit number (as categorical factors) were included as fixed effects, while the subject was treated as a random effect. Furthermore, changes in FEV1 over 12 months post-randomization were evaluated in a two-step process. Initially, changes for each patient were determined by linear regression of five FEV1 measurements, assuming the slope represented the change over time. Then, these slopes were analyzed based on treatment groups and genotype, with *p*-values adjusted for multiple comparisons using the Dunnett–Hsu method. The overall effect of treatment across polymorphisms was calculated. Health-related QoL improvement over 12 months was assessed using generalized linear models, examining the impact of treatment and genotype. Regression and mixed-effects models were adjusted for relevant clinical covariates (age, sex, baseline eosinophil count, smoking status [pack-years], and baseline FEV_1_) to minimize potential confounding. Deviations in genotype frequencies from the Hardy–Weinberg equilibrium were checked using the χ2 goodness-of-fit test, and comparisons of genotype and allele distributions were performed using the χ2 test of independence. An alpha error threshold of 0.05 was set for statistical significance. Analyses were performed using the Statistical Analysis System (SAS^®^ Institute, Cary, NC, USA), IBM SPSS Statistics (Version 28), and RStudio Team (Version 2021.09.0+351; Ghost Orchid).

## 5. Conclusions

In this study, we provide evidence that the rs793391 polymorphism in *SUMF1* may be associated with differential response to ICS treatment, in particularly budesonide, in COPD patients receiving LAMA/LABA. Heterozygous carriers (TG) showed a favorable response to ICS when added to LAMA/LABA, as indicated by a significant improvement in FEV_1_ over 12 months. These patients also experienced a reduction in SGRQ total scores, along with improvements in CAT and General Health scores, compared to those receiving LAMA/LABA/placebo. This is the first study to link *SUMF1* genetic variants to treatment response in a well-characterized COPD cohort within the setting of a randomized controlled trial. While promising, these findings should be considered exploratory and hypothesis-generating, warranting validation in larger, independent cohorts.

## Figures and Tables

**Table 1:** Baseline demographic and clinical characteristics of 165 COPD patients included in the genotype analysis.

Sex	
Male	114 (69.1)
Female	51 (30.9)
Age in years	67 (59–72)
Ethnic origin	
White	163 (100)
Smoking status	
Former smokers	103 (62.4)
Current smokers	62 (37.6)
Pack Years	50 (37–70)
Disease duration (months)	60 (23–120)
**Disease characteristics at baseline**	
Post-bronchodilator FEV_1_ (liters)	1.59 (1.2–2.0)
Post-bronchodilator FEV_1_ (% of predicted value)	59 (49–67)
Post-bronchodilator DLCO_SB (% of predicted value)	61.5 (48.4–78.2)
Post-bronchodilator TLC (% of predicted value)	109 (98–122)
Post-bronchodilator RV (% of predicted value)	136 (111–162)
Post-bronchodilator ratio RV/TLC (% of predicted value)	116 (104–133)
Fractional exhaled NO (ppb)	15 (8–23)
6 min walking distance (meters)	460 (390–524)
MMRC dyspnea scale	1 (0–2)
CAT score, total sum	14 (9–19)
SGRQ, total score	32.8 (20.0–48.7)
ASMC (%) *	17.4 (12.4–22.2)

Data are *n* (%) or median (IQR). FEV1 = forced expiratory volume in 1 sec; DLCO = diffusing capacity for carbon monoxide; TLC = total lung capacity; RV = residual volume; NO = nitric oxide; MMRC = Modified Medical Research Council Dyspnoea Scale; CAT= COPD Assessment Test; SGRQ = Saint George Respiratory Questionnaire; ASMC = airway smooth muscle cells. * ASMC were assessed in endobronchial biopsies (N = 10) obtained from the following anatomic locations: right upper lobe (*n* = 2), right middle lobe (*n* = 2), right lower lobe (*n* = 2), left upper lobe, (*n* = 2), left lower lobe (*n* = 2). Five sequential sections were prepared from each biopsy, stained with hematoxylin and eosin and elastica van Gieson and evaluated for ASMC mass as % of the total are of bronchial tissue (excluding any cartilage that may have been present in the biopsy). Numbers represent median values (IQR) from the mean values of ASMC (%) obtained from all biopsies.

**Table 2:** Genotype distribution and allele frequencies of rs11915920 and rs793391 among the 165 COPD patients included in the genotype analysis.

SNP	Chromosome Position	Genotype Frequencies *n* (%)	Minor Allele	MAF %	HWE (*p*)
rs11915920 C > T	chr3:4368850	CC = 33 (20) CT = 83 (50.3) TT = 49 (29.7)	C	45.2	0.84
	Intron Variant				
rs793391 T > G	chr3:4427508	TT = 91 (55.2) TG = 65 (39.4) GG = 9 (5.5)	G	25.1	0.55
	Intron Variant				

Genetic variations were identified and cross-referenced using the Single Nucleotide Polymorphism database (dbSNP, version 156) maintained by the National Center for Biotechnology Information (NCBI). HWE: Hardy–Weinberg equilibrium; MAF: minor allele frequency.

**Table 3:** Differences in CAT score values over 12 months between treatment groups stratified according to rs11915920 and rs793391 *SUMF1* genotypes.

SNP	Genotype	Treatment Group (*n*)	Mean Change Score (Min–Max) in 12 Months	Difference Between Treatment Groups (Units)	*p*-Value
rs793391	TT	LAMA/LABA/placebo (40)	−2.05	1.50	0.66
		LAMA/LABA/ICS (41)	−0.55		
	TG	LAMA/LABA/placebo (27)	0.70	−2.68	0.17
		LAMA/LABA/ICS (35)	−1.97		
	GG	LAMA/LABA/placebo (4)	3.75	−0.35	0.92
		LAMA/LABA/ICS (5)	3.40		
rs11915920	CC	LAMA/LABA/placebo (15)	1.40	−1.15	0.89
		LAMA/LABA/ICS (16)	0.25		
	CT	LAMA/LABA/placebo (35)	−1.03	0.27	0.76
		LAMA/LABA/ICS (44)	−0.76		
	TT	LAMA/LABA/placebo (25)	−1.64	−0.50	0.58
		LAMA/LABA/ICS (21)	−2.14		

LAMA: long-acting muscarinic antagonists; LABA: long-acting beta-2 agonists; ICS: inhaled corticosteroids. A difference of 2 or more units in the CAT score suggests a clinically relevant change in health status.

**Table 4:** Differences in SF-36 General Health score values in 12 months between treatment groups stratified according to rs11915920 and rs793391 *SUMF1* genotypes.

SNP	Genotype	Treatment Group (*n*)	Mean Change Score (Min–Max) in 12 Months	Difference Between Treatment Groups (Units)	*p*-Value
rs793391	TT	LAMA/LABA/placebo (40)	−6.38 (−40–30)	2.94	0.75
		LAMA/LABA/ICS (41)	−3.44 (−95–27.5)		
	TG	LAMA/LABA/placebo (27)	−11.40 (−45–30)	8.12	0.39
		LAMA/LABA/ICS (35)	−3.28 (−40–30)		
	GG	LAMA/LABA/placebo (4)	5.00 (−5–20)	−24.00	0.018
		LAMA/LABA/ICS (5)	−19.00 (−40–5)		
rs11915920	CC	LAMA/LABA/placebo (15)	−5.67 (−45–30)	9.00	0.34
		LAMA/LABA/ICS (16)	3.33 (−35–30)		
	CT	LAMA/LABA/placebo (35)	−5.78 (−40–30)	−0.85	0.93
		LAMA/LABA/ICS (44)	−6.63 (−95–25)		
	TT	LAMA/LABA/placebo (25)	−11.36 (−40–20)	5.97	0.52
		LAMA/LABA/ICS (21)	−5.40 (−40–30)		

LAMA: long-acting muscarinic antagonists; LABA: long-acting beta-2 agonists; ICS: inhaled corticosteroids. The SF-36 General Health measure consists of five items. The lower the score, the more prevalent the disability. Differences of ≥3–5 points in the SF-36 scales have been proposed as a clinically relevant or minimum clinically important difference.

## Data Availability

Individual participant data will not be made available. Protocol details may be available upon request to corresponding author.

## Supplementary Materials

The following supporting information can be downloaded at: https://www.mdpi.com/article/10.3390/ijms262010225/s1.

## Abbreviations

The following abbreviations are used in this manuscript:

ACL	Aclidinium
AECOPD	Acute Exacerbations of COPD
ASMC	Airway Smooth Muscle Cells
BAL	Bronchoalveolar Lavage
BUD	Budesonide
CAT	COPD Assessment Test
COPD	Chronic Obstructive Pulmonary Disease
CT	Computed Tomography
DLCO	Diffusing Capacity for Carbon Monoxide
ECM	Extracellular Matrix
FEV_1_	Forced Expiratory Volume in 1 Second
FOR	Formoterol
GAGs	Glycosaminoglycans
GOLD	Global Initiative for Chronic Obstructive Lung Disease
GWAS	Genome-Wide Association Study
HWE	Hardy–Weinberg Equilibrium
ICS	Inhaled Corticosteroids
ISRCTN	International Standard Randomized Controlled Trial Number
LABA	Long-Acting Beta-2 Agonist
LAMA	Long-Acting Muscarinic Antagonist
LD	Linkage Disequilibrium Analysis
MAF	Minor Allele Frequency
MCID	Minimal Clinically Important Difference
MMRC	Modified Medical Research Council (Dyspnea Scale)
NCBI	National Center for Biotechnology Information
NO	Nitric Oxide
QoL	Quality of Life
RCT	Randomized Controlled Trial
RNA	Ribonucleic Acid
RV	Residual Volume
SABA	Short-Acting Beta-2 Agonist
SAMA	Short-Acting Muscarinic Antagonist
SAS^®^	Statistical Analysis System
SGRQ	St. George’s Respiratory Questionnaire
SF-36	Short Form-36 (General Health Questionnaire)
SNP	Single Nucleotide Polymorphism
SUMF1	Sulfatase Modifying Factor 1
TLC	Total Lung Capacity
