# Supplementary material for: SUMF1 Common Variant rs793391 Is Associated with Response to Inhaled Corticosteroids in Patients with COPD
Source: Int J Mol Sci. 2025 Oct 21;26(20):10225. doi: 10.3390/ijms262010225 (PMC12564353; doi:10.3390/ijms262010225)
Supplement: Supplementary file 1 [file ijms-26-10225-s001.zip › ijms-3816912-supplementary.pdf]

Supplementary Material: *SUMF1* common variant rs793391 is associated with response to inhaled corticosteroids in patients with COPD

Table S1. Baseline additional laboratory and clinical characteristics of 165 COPD patients included in the genotype analysis	
CT scan performed	164 (99.4)
Emphysema in CT scan	130 (83.3)
Eosinophils	
Blood eosinophils (x 10^9/l)	0.16 (0.1-0.2)
BAL eosinophils	1 (0.7-2.0)
Tissue eosinophilic infiltration*	
Absence	117 (72.7)
Mild-Moderate	43 (26.7)
Severe	1 (0.6)
AECOPD in previous year	
Urgent physician visit	119 (75.8)
Ambulatory	100 (63.7)
Hospitalization	41 (24.8)
Intensive care	7 (4.2)
Pre-study Medication	
No medication	16 (9.7)
Only SABA	1 (0.6)
Only LABA	2 (1.2)
Only LAMA	10 (6.1)
SABA + LAMA	1 (0.6)
SABA + LABA/LAMA	3 (1.8)
SABA + LABA/ICS	5 (3.0)
SABA + LAMA/ICS	1 (0.6)
SABA + LABA/LAMA/ICS	11 (6.7)
SAMA + ICS	1 (0.61)
SAMA + LABA/ICS	1 (0.6)
SAMA + LABA/LAMA	1 (0.6)
SAMA + LABA/LAMA/ICS	1 (0.6)
SABA/SAMA + LABA/LAMA	2 (1.2)

SABA/SAMA + LABA/LAMA/ICS	3 (1.8)
LABA/ICS	13 (7.9)
LABA/LAMA	41 (24.9)
LABA/LAMA/ICS	51 (30.9)
Only mucolytics/antioxidant	1 (0.6)
Oral steroids	6 (3.6)
Oxygen Therapy	20 (12.1)

Data are n (%) or median (IQR) ASMC: airway smooth muscle; AECOPD: acute exacerbations of COPD; LABA=long-acting beta-2 agonists; LAMA=long-acting muscarinic antagonists; SABA=short acting beta-2 agonists; ICS=inhaled corticosteroids; BAL=Bronchoalveolar Lavage; ASMC=Airway Smooth Muscle Cells
*Tissue eosinophilic infiltration in endobronchial biopsies was assessed qualitatively using a 0-3 scale: 0 = absence / normal, 1 = mild-moderate, 2-3= severe
**ASMC were assessed in endobronchial biopsies (N=10) obtained from the following anatomic locations: right upper lobe (n=2), right middle lobe (n=2), right lower lobe (n=2), left upper lobe, (n=2), left lower lobe (n=2). Five sequential sections were prepared from each biopsy, stained with hematoxylin and eosin and elastica van Gieson and evaluated for ASMC mass as % of the total are of bronchial tissue (excluding any cartilage that may have been present in the biopsy). Numbers represent median values (IQR) from the mean values of ASMC (%) obtained from all biopsies.

Supplementary Table 2. Lung function parameters following bronchodilation at baseline across the different genotypes of rs11915920 and rs793391 of the *SUMF1* gene

**Lung Function parameter Median (IQR)**	**rs11915920**			
	**CC**	**CT**	**TT**	p-value
FRCpleth (% predicted)	127 (104 - 156)	140 (121 - 160)	129 (111 - 153)	0.09
RV (% predicted)	129 (105 - 157)	138 (112 - 168)	137 (116 - 159)	0.28
FVC (% predicted)	92 (83 - 103)	91 (79 - 104)	91 (80 - 106)	0.86
TLC (% predicted)	104 (93 - 120)	112 (99 - 123)	107 (98 - 117)	0.28
FEV1 (liters)	1.69 (1.23 - 2.05)	1.59 (1.17 - 1.89)	1.58 (1.20 - 2.10)	0.57
FEV1 (% predicted)	61 (52 - 67)	57 (49 - 67)	62 (45 - 70)	0.55
DLCO_SB (% predicted)	63.5 (47.1 - 80.2)	56.7 (46.5 - 71.5)	65.6 (51.9 - 82.6)	0.08
N2-SBW (% predicted)	596 (465 - 744)	640.5 (482 - 848)	610 (458 - 950)	0.64
	**rs793391**			
	**TT**	**TG**	**GG**	p-value
FRCpleth (% predicted)	136 (116 - 156)	138 (115 - 160)	114 (101 - 158)	0.53
RV (% predicted)	137 (113 - 160)	136 (107 - 165)	133 (111 - 157)	0.96
FVC (% predicted)	91 (79 - 106)	93 (83 - 105)	83 (71 - 88)	0.20
TLC (% predicted)	109 (98 - 122)	111 (97 - 122)	103 (95 - 120)	0.76
FEV1 (liters)	1.6 (1.2 - 2)	1.6 (1.2 - 2)	1.9 (1.7 - 2.1)	0.30
FEV1 (% predicted)	57 (48 - 70)	59 (50 - 66)	63 (53 - 67)	0.92

DLCO_SB (% predicted)	61.2 (45.6 - 79.3)	62.5 (50.8 - 75.1)	63 (49.7 - 80.2)	0.96
N2-SBW (% predicted)	610 (465 - 826)	639 (494 - 930)	662 (451 - 815)	0.44

Results presented as medians and interquartile ranges.
FRC: Functional residual capacity measured by plethysmography; RV: Residual volume; FVC: Forced vital capacity; FEV1: Forced expiratory volume in 1 second; TLC: Total lung capacity; DLCO SB: Diffusing capacity of the lungs for carbon monoxide single breath; DLCO SB/VA Diffusing capacity of the lungs for carbon monoxide single breath per liter of alveolar volume; SIII = N2 single breath washout phase III slope

Supplementary Table 3. Histological characteristics evaluated in endobronchial biopsies stratified according to rs11915920 and rs793391 *SUMF1* genotypes.

Histological Characteristic		rs11915920			p-value
		**CC**	**CT**	**TT**	
Inflammation in the stroma n (%)	no	0 (0.0)	0 (0.0)	0 (0.0)	0.82
	mild	20 (80)	38 (69.09)	31 (73.80)	
	moderate	5 (20)	16 (29.09)	11 (26.19)	
	severe	0 (0)	1 (1.82)	0 (0)	
Tissue lymphocyte infiltration n (%)	no	7 (21.87)	27 (33.75)	7 (14.28)	0.06
	mild	22 (68.75)	38 (47.5)	33 (67.34)	
	moderate	3 (9.38)	15 (18.75)	9 (18.36)	
	severe	0 (0·0)	0 (0·0)	0 (0·0)	
Tissue eosinophil infiltration n (%)	no	24 (75)	56 (70)	26 (53.06)	**0.006**
	mild	3 (9.38)	20 (25)	10 (20.40)	
	moderate	5 (15·62)	3 (3.75)	12 (24.48)	
	severe	0 (0)	1 (1.25)	1 (2.04)	
Tissue neutrophil infiltration n (%)	no	26 (81.25)	67 (83.75)	36 (73.46)	0.15
	mild	4 (12.5)	13 (16.25)	11 (22.44)	
	moderate	2 (6.25)	0 (0)	2 (4.08)	
	severe	0 (0.0)	0 (0.0)	0 (0.0)	
Basement membrane thickening n (%)	no	0 (0)	1 (1.25)	2 (4.08)	0.30
	mild	13 (40.62)	29 (36.25)	16 (32.65)	
	moderate	18 (56.25)	36 (45)	22 (44.89)	
	severe	1 (3.13)	14 (17.5)	9 (18.36)	
ASMC %	n	29	69	38	**0.015**
	Median (IQR)	19.33 (14.38-28.25)	15.75 (10.63-21.00)	20.65 (14.38 - 27.75)	
		**rs793391**			p-value
		**TT**	**TG**	**GG**	
Inflammation in the stroma n (%)	no	0 (0.0)	0 (0.0)	0 (0.0)	0.82
	mild	52 (74.28)	32 (69.56)	5 (83.33)	
	moderate	17 (24.28)	14 (30.43)	1 (16.66)	
	severe	1 (1.43)	0 (0)	0 (0)	
Tissue lymphocyte infiltration n (%)	no	18 (20.45)	20 (31.25)	3 (33.33)	0.32
	mild	57 (64.77)	31 (48.43)	5 (55.55)	
	moderate	13 (14.77)	13 (20.31)	1 (11.11)	
	severe	0 (0.0)	0 (0.0)	0 (0.0)	
Tissue eosinophil infiltration n (%)	no	54 (61.36)	46 (71.87)	6 (66.66)	0.84
	mild	20 (22.72)	11 (17.18)	2 (22.22)	
	moderate	13 (14.77)	6 (9.38)	1 (11.11)	
	severe	1 (1.14)	1 (1.56)	0 (0)	
	no	70 (79.54)	53 (82.81)	6 (66.66)	0.05
	mild	18 (20.45)	8 (12.5)	2 (22.22)	

Tissue neutrophil infiltration n (%)	moderate	0 (0)	3 (4.69)	1 (11.11)	
	severe	0 (0.0)	0 (0.0)	0 (0.0)	
Basement membrane thickening n (%)	no	2 (2.27)	1 (1.56)	0 (0)	0.32
	mild	26 (29.54)	26 (40.62)	6 (66.66)	
	moderate	44 (50)	30 (46.87)	2 (22.22)	
	severe	16 (18.18)	7 (10.93)	1 (11.11)	
ASMC %	n	77	52	7	0.81
	Median (IQR)	17.88 (13.75-24.17)	17.60 (12.40-24.19)	20.29 (13.13-25.00)	
Results expressed as n (%).					

**Supplementary Table 4** Number of blood cells stratified according to different genotypes of the rs11915920 and rs793391 SUMF1 polymorphisms				
**Blood cell type**	**rs11915920**			
	**CC**	**CT**	**TT**	p-value
	n = 33	n = 83	n = 49	
Eosinophils (10^9/L)	0.18 (0.13 to 0.30)	0.16 (0.10 to 0.25)	0.16 (0.09 to 0.22)	0.29
Eosinophils (%)	2.9 (1.5 to 4.2)	2.2 (1.3 to 3.2)	2.4 (1.3 to 3)	0.29
Leukocytes	7.53 (6.01 to 9.39)	7.47 (6.67 to 8.72)	7.31 (6.14 to 8.78)	0.80
Lymphocytes (10^9/L)	1.87 (1.49 to 2.20)	1.62 (1.22 to 2.14)	1.69 (1.29 to 1.99)	0.07
Lymphocytes (%)	25.3 (20 to 32.5)	21.1 (17.2 to 27.6)	23.3 (18.7 to 28.0)	0.07
Neutrophils (10^9/L)	4.42 (3.64 to 6.18)	4.99 (4.2 to 5.9)	4.93 (3.87 to 5.61)	0.58
Neutrophils (%)	61.7 (54.7 to 65.3)	66.4 (59.8 to 73.1)	64.3 (57.4 to 71.8)	**0.013**
**Blood cell type**	**rs793391**			
	**TT**	**TG**	**GG**	p-value
	n = 91	n = 65	n = 9	
Eosinophils (10^9/L)	0.27 (0.22 to 0.35)	0.16 (0.11 to 0.25)	0.15 (0.09 to 0.24)	**0.050**
Eosinophils (%)	3.4 (2.1 to 5.1)	2.3 (1.3 to 3.2)	2.2 (1.3 to 3.1)	0.28
Leukocytes	9.39 (6.83 to 9.98)	7.53 (6.63 to 8.72)	7.44 (6.12 to 8.74)	0.24
Lymphocytes (10^9/L)	1.83 (1.38 to 2.25)	1.67 (1.30 to 2.14)	1.678 (1.26 to 2.00)	0.72
Lymphocytes (%)	22.5 (16.5 to 29.3)	22.2 (17.2 to 29.1)	22.6 (18.7 to 28.1)	0.96
Neutrophils (10^9/L)	6.131 (4.29 to 6.76)	4.88 (3.95 to 5.61)	4.9 (3.73 to 5.89)	0.48
Neutrophils (%)	62.9 (57.2 to 67.9)	65.3 (56.8 to 72.5)	65.1 (59.1 to 71.4)	0.81
Data are presented as n, median (interquartile range (IQR))				

Supplementary Table 5. Adjusted annual exacerbation rates (AECOPD/year) by genotype and treatment group

Treatment Group	Genotype	Adjusted Mean (95% CI)	p-value vs. TG
		**rs793391**	
LAMA/LABA/Placebo	TT	0.867 (0.554–1.180)	0.711
	TG	1.051 (0.652–1.450) *(Ref)*	–
	GG	0.455 (–0.582–1.492)	0.483
LAMA/LABA/ICS	TT	0.763 (0.478–1.048)	0.720
	TG	0.613 (0.304–0.922) *(Ref)*	–
	GG	1.670 (0.853–2.486)	0.036
		**rs11915920**	
LAMA/LABA/Placebo	CC	0.850 (0.312–1.388)	0.999
	CT	0.842 (0.489–1.194) *(Ref)*	–
	TT	1.046 (0.629–1.463)	0.691
LAMA/LABA/ICS	CC	1.042 (0.577–1.507)	0.491
	CT	0.754 (0.473–1.034) *(Ref)*	–
	TT	0.536 (0.130–0.942)	0.607

Values are least squares means (LS mean) with 95% confidence intervals (CI). Descriptive statistics of the annual number of acute exacerbations of COPD (AECOPD/year) were calculated according to genotypes of rs793391 and rs11915920. Associations between genotype and AECOPD frequency were assessed separately for the two treatment groups (LAMA/LABA/placebo and LAMA/LABA/ICS) using generalized linear models. Multiple comparisons were adjusted using the Dunnett–Hsu method.

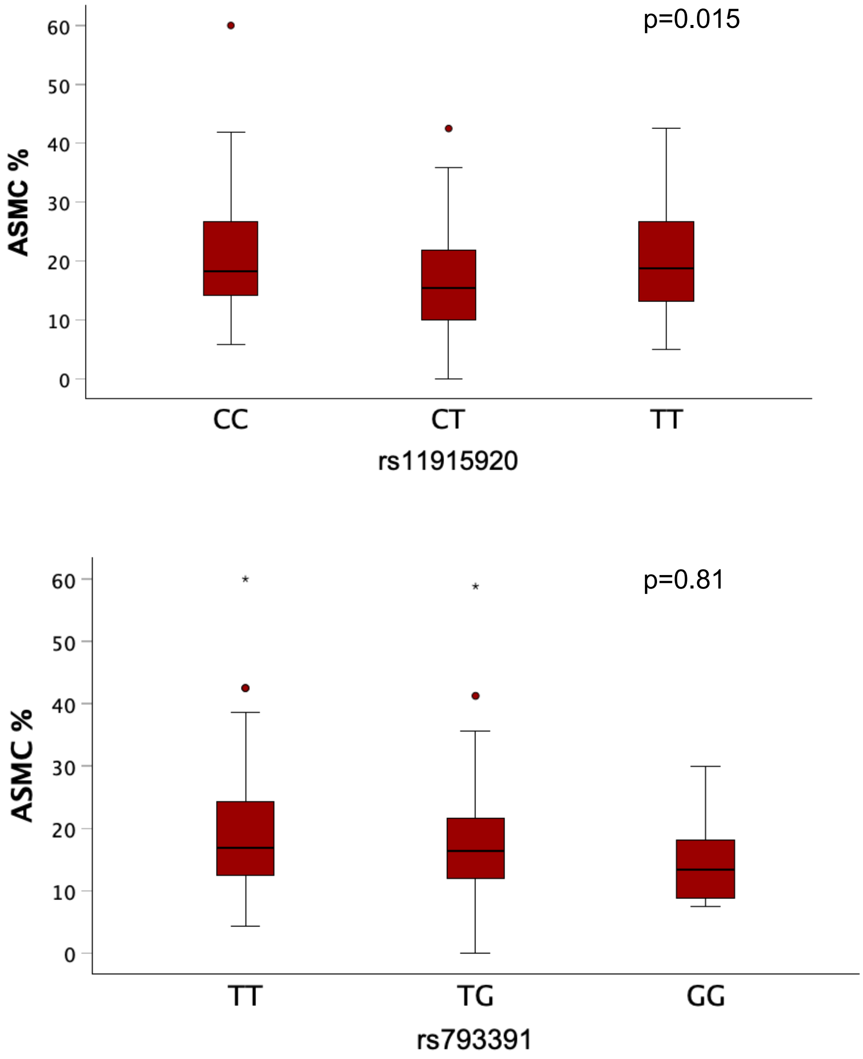

Supplementary Figure 1. ASMC % distribution according to the different genotypes of rs11915920 and rs793391. ASMC: airway smooth muscle cell area; ASMCs were assessed in endobronchial biopsies (n=10) obtained from the following anatomic locations: right upper lobe (n=2), right middle lobe (n=2), right lower lobe (n=2), left upper lobe, (n=2), left lower lobe (n=2). Five sequential sections were prepared from each biopsy, stained with haematoxylin and eosin and elastica Van Gieson and evaluated for ASMC area as percentage of the total area of bronchial tissue (excluding any cartilage that may have been present in the biopsy). Box-plots represent the 25th–75th percentile. Median values are shown with a horizontal line within each box and whiskers indicate the 5th–95th percentile range. Genotypes presented with the reference/reference genotype to the left.

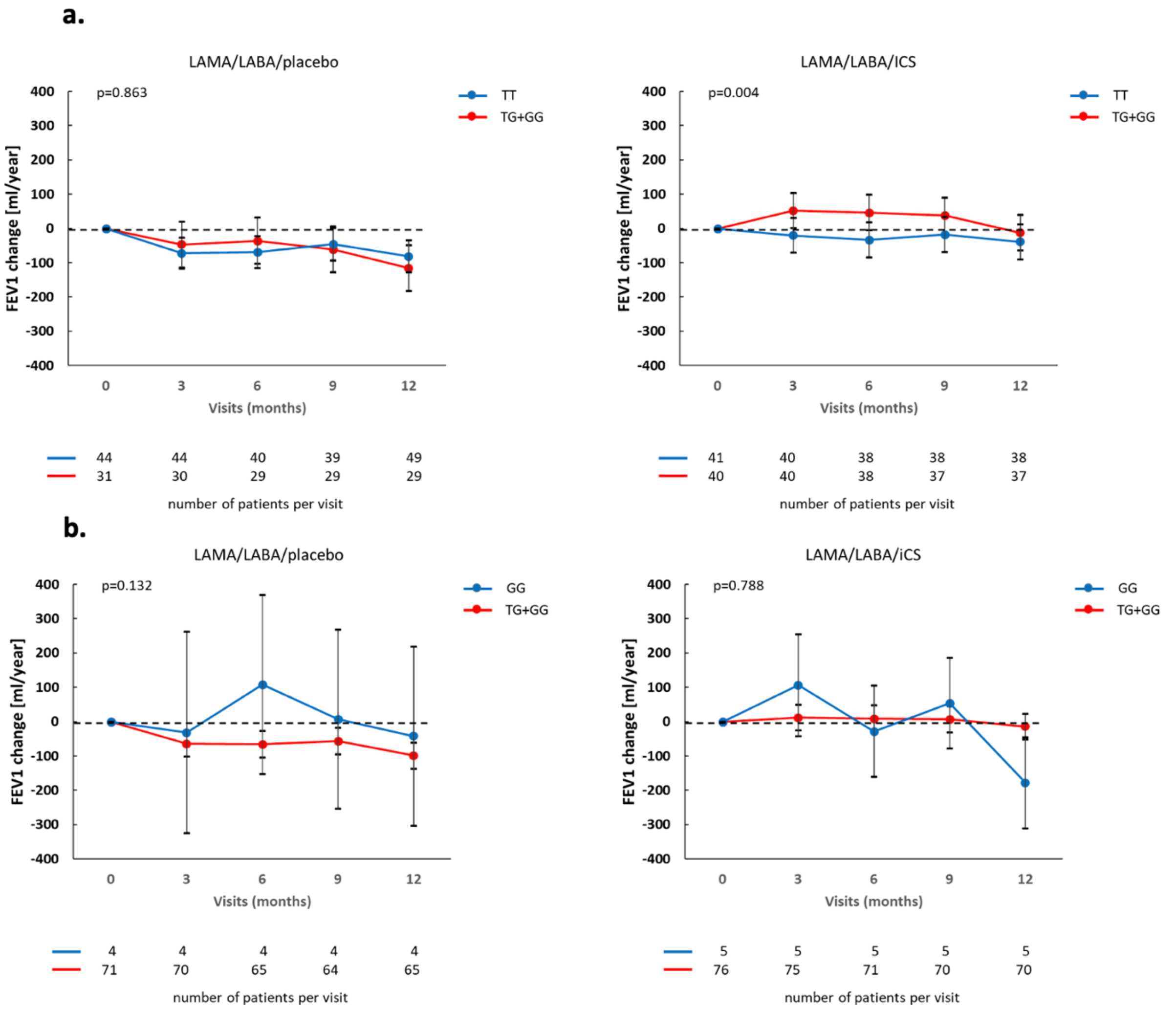

Supplementary Figure 2. FEV_1 change (mL) over the study visits during 12 months for patients randomized to LAMA/LABA/placebo and LAMA/LABA/ICS by rs793391 genotype, using (a) the dominant model (TT vs. TG+GG) and (b) the recessive model (GG vs. TT+TG). The association between FEV_1 change from visit 1 in the treatment groups and different visits was evaluated using a mixed-effects model. The treatment group and visit number (categorical factors) were included as fixed effects and the subject as a random effect. The Dunnett–Hsu method was used to correct p-values for multiple comparisons. To calculate the change from visit 1 to visit 5 for each treatment group, an analysis of covariance was used.

a. dominant model   b. recessive model

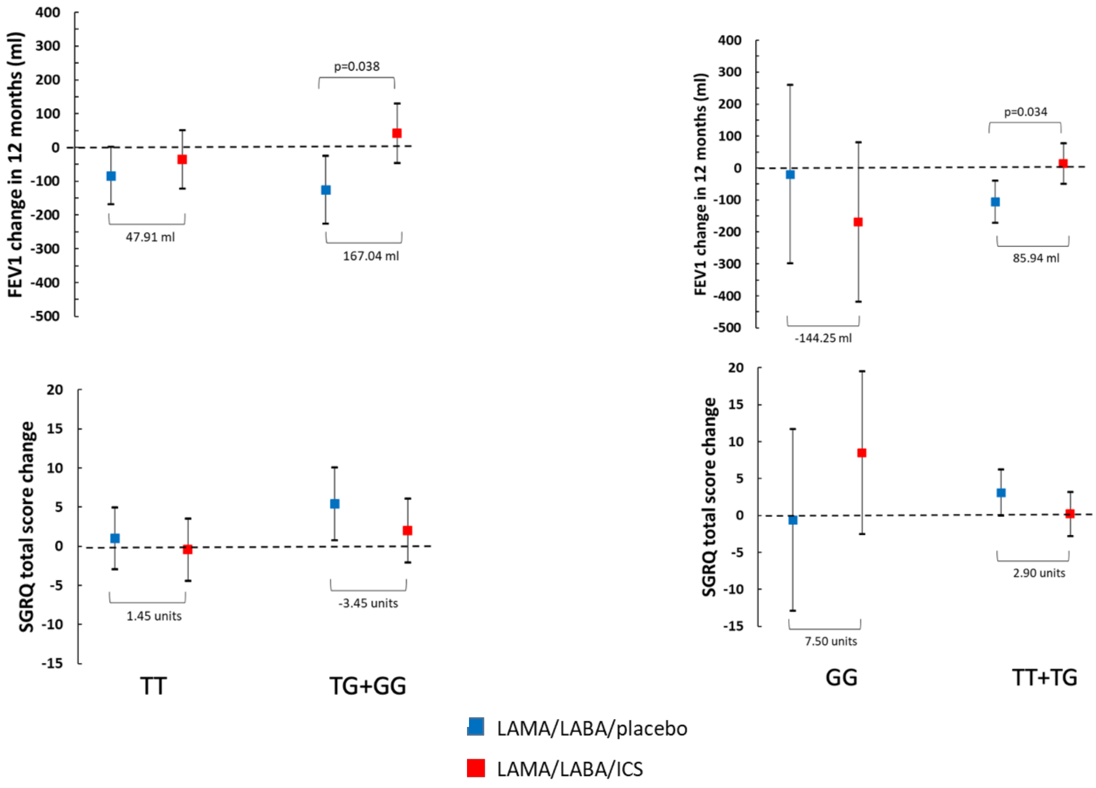

Supplementary Figure 3. Change in FEV_1 (mL) and SGRQ score over 12 months in COPD patients randomized to LAMA/LABA/placebo or LAMA/LABA/ICS, stratified by rs793391 genotype according to (a) the dominant model (TT vs. TG+GG) and (b) the recessive model (GG vs. TT+TG). The squares represent the adjusted mean values and the whiskers indicate the 95% confidence intervals.

A two-step analysis was used to evaluate the change in FEV_1 from the first visit (after a 6-week run-in period with triple therapy) to the last visit (12 months after randomization). In the first step, the FEV_1 change in each patient was estimated as the slope of a linear regression of the five FEV_1 measurements over time. In the second step, these slopes were regressed against genotype-treatment groups.

A generalized linear regression model was used to evaluate the change in SGRQ total score from the first to the last visit. Total scores on the SGRQ range from 0 to 100, with lower scores indicating better health-related quality of life. A decrease in SGRQ score of ≥4 units indicates a clinically meaningful improvement. The Dunnett–Hsu method was used to correct p-values for multiple comparisons. Only statistically significant p-values are shown.

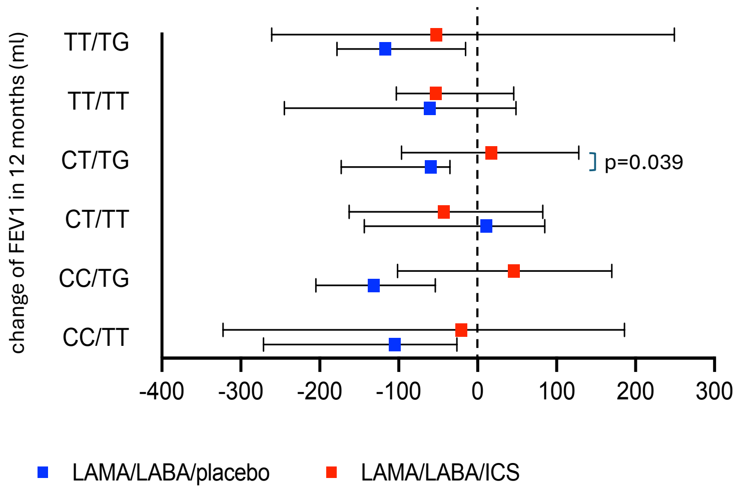

Supplementary Figure 4. Visualization of the change of forced expiratory volume in 1 s (FEV1) (mL) in 12 months in COPD patients with rs11915920 and rs793391 genotypes combination (rs11915920/rs793391). The squares represent the mean and the whiskers the 95% intervals. Two step regression models were used to evaluate the change of FEV1 from the first visit (visit 1 – after 6 weeks run-in period with triple therapy) to the last visit, 12 months after randomization, in the treatment groups. In the first step, the FEV1 change in each patient was calculated as the slope of a linear regression of the five FEV1 measurements over time. In the second step, the slopes representing the change in FEV1 were regressed in function of the treatment groups. Only subgroups of ≥5 patients per treatment are presented. Only statistically significant p-values are shown.
